# Sex-differences in the multidimensional evaluation of dyspnea in respiratory outpatients

**DOI:** 10.3389/fmed.2025.1627496

**Published:** 2025-07-09

**Authors:** Kathryn M. Milne, Julia Zhang, Owen D. Harris, Jordan A. Guenette

**Affiliations:** ^1^Centre for Heart Lung Innovation, St. Paul’s Hospital, The University of British Columbia and Providence Research, Vancouver, BC, Canada; ^2^Division of Respiratory Medicine, The University of British Columbia, Vancouver, BC, Canada; ^3^Centre for Lung Health, The University of British Columbia, Vancouver, BC, Canada; ^4^Department of Physical Therapy, The University of British Columbia, Vancouver, BC, Canada

**Keywords:** anxiety, dyspnea, sex-differences, depression, chronic lung disease

## Abstract

**Background:**

Females experience greater dyspnea intensity compared to males. The role of sex in dyspnea quality and emotional responses in respiratory outpatients is unknown. The aim of this study was to examine sex-differences in dyspnea quality and affective distress as well as relationships between dyspnea, anxiety, and depression.

**Materials and methods:**

Respiratory outpatients rated chronic exertional dyspnea intensity and impact using the modified Medical Research Council (mMRC) dyspnea scale. Participants selected qualitative dyspnea descriptors from an established list of 15 descriptors and completed the Multidimensional Dyspnea Profile (MDP). Symptoms of anxiety and depression were assessed using the Hospital Anxiety and Depression Scale (HADS).

**Results:**

Participants (*n* = 78) had a variety of common respiratory diagnoses. Males and females experienced similar dyspnea impact (mMRC) and were matched for relative impairment in lung function. Females rated higher intensity of breathing muscle work, chest tightness, and mental effort. The affective response to dyspnea was greater in females, with significantly higher reports of anxiety, frustration, and fear related to dyspnea. HADS anxiety subscale scores were correlated with MDP dyspnea scores of breathing discomfort, immediate perception domain, and emotional response domain in males. In females, higher HADS anxiety scores were correlated with the emotional response domain only. Males with higher likelihood of anxiety experienced greater frustration, anger, and fear related to dyspnea; however, this was not the case for females.

**Conclusion:**

In respiratory outpatients, despite similar lung function impairment and mMRC scores, females experience greater breathing muscle work, chest tightness, and mental effort as well as significantly greater anxiety, frustration, and fear related to dyspnea.

## 1 Introduction

Dyspnea is a multidimensional subjective experience of breathing discomfort comprised of distinct but interconnected domains: symptom impact, sensory-perceptual, and affective distress (1). Breathlessness is a significant predictor of morbidity and mortality in the general population and can have a debilitating impact on quality of life (1). Healthy females and those with chronic respiratory diseases experience greater dyspnea intensity compared to males (2–6). Both detailed exercise physiology studies and population-based studies have demonstrated that differences in absolute lung function and relative ventilatory capacity during exercise underpin the greater burden of exertional dyspnea intensity experienced by females (2, 3, 6–11). However, the potential role of sex in dyspnea quality and emotional responses in a real-world respiratory outpatient setting is unknown.

Differences in the sensory-perceptual domain of dyspnea quality were described by Simon et al. in two landmark studies (12, 13). Dyspnea quality varied in healthy participants across different experimental conditions used to induce dyspnea (12), and unique combinations of dyspnea quality descriptors were associated with different diagnoses in participants with chronic disease (13). Subsequent to these findings, various validated and reliable tools have been developed for assessment of dyspnea quality as well as associated affective distress (14–16). For example, the multidimensional dyspnea profile (MDP) is a validated and reliable tool for assessment of dyspnea in healthy and clinical populations that includes assessment of dyspnea unpleasantness, quality, and emotional response (16).

Previous work suggests that females are more likely to select qualitative dyspnea descriptors related to unsatisfied inspiration and shallow breathing relative to males at peak exercise; however, this was limited to younger healthy adults (7). In cardiorespiratory disease populations, studies have assessed differences in dyspnea quality compared to health, but potential sex-differences in dyspnea quality have not been comprehensively explored (13, 15, 17–21). Assessment of sex-differences in the affective response to dyspnea in respiratory outpatients presenting for assessment by a physician has not previously been described. It is well-established that there is a high prevalence of anxiety and depression in patients with chronic dyspnea (1, 22–25). However, the combined impact of anxiety, depression, and sex on dyspnea ratings in the outpatient respirology setting remains to be determined. Accordingly, the primary aim of this cross-sectional study was to examine sex-differences in dyspnea quality and affective distress in respiratory outpatients. Our secondary aim was to assess relationships between dimensions of dyspnea, anxiety, and depression.

## 2 Materials and methods

### 2.1 Study design and participants

This cross-sectional study recruited outpatients presenting to the Center for Lung Health and Vancouver General Hospital respirology clinics. Patients were invited to participate in the study at clinic visits or through a written letter of initial contact. Included patients were referred for assessment of dyspnea or had a history of dyspnea, 18 years of age or older, able to complete study questionnaires in English, and able to provide informed consent. Patients hospitalized within 90 days of study participation, those with previous thoracic surgery, and lung transplant recipients were excluded. Male and female sex was defined as sex assigned at birth. Each participant completed study questionnaires only once during the study period. This study was approved by The University of British Columbia and Providence Health Care Research Institute Ethics Board. All participants provided written informed consent.

### 2.2 Study procedures

Study visits were conducted in-person or virtually (Zoom, Zoom Video Communications Inc.) based on participant preference. Participants completed dyspnea questionnaires anchored to the most severe episode of dyspnea experienced in the 6 weeks prior to the study visit. Chronic exertional dyspnea intensity and impact were assessed using the modified Medical Research Council (mMRC) dyspnea scale and Baseline Dyspnea Index (BDI) (26, 27). Participants also characterized their breathlessness by selecting qualitative dyspnea descriptors from an established list of 15 descriptors (14) and completed the MDP (16).

The MDP assesses multiple components of dyspnea including breathing discomfort, sensory qualities of dyspnea, and emotional response. These aspects are assessed individually and grouped into dimensions and domains (16). The sensory dimension encompasses sensory qualities of dyspnea, while the affective dimension includes breathing discomfort and emotional responses. The immediate perception domain comprises breathing discomfort and the sensory qualities, whereas the emotional response domain focuses on emotional responses (16). MDP scoring provides values for breathing discomfort, immediate perception, and emotional response domains, with sensory qualities and affective responses also scored separately (16).

Symptoms of anxiety and depression were assessed using the Hospital Anxiety and Depression Scale (HADS) (28). A threshold equal to or greater than 8 for anxiety or depression subscale scores was used to define participants with a higher likelihood of anxiety or depression (28). Participants rated their comfort discussing feelings of anxiety with a physician using a visual analogue scale (VAS). Using this scale, 0 represented total discomfort and 100 represented total ease discussing feelings of anxiety. Demographic, anthropomorphic, medical history, and pulmonary function data were extracted from the medical record.

### 2.3 Statistical analysis

Independent samples *t*-tests and Fisher’s exact tests were used to evaluate between group differences in questionnaire responses. Pearson’s correlation was used to assess relationships between MDP and HADS scores. Multiple linear regression was used to determine the relationship between dyspnea and sex, age, body mass index, and smoking status, as long as standard assumptions for multiple linear regression were met. Statistical significance was defined as *p* < 0.05. Presented values are mean ± standard deviation unless otherwise specified. Statistical analyses were performed in SPSS (SPSS v29, IBM).

## 3 Results

### 3.1 Study participants

Recruitment for the study took place between June 2021 and May 2022. A total of 78 participants took part in the study and completed study questionnaires. Anthropomorphic and demographic data are presented in Table 1. More males were ex-smokers compared to females (proportion male smokers 27/42 vs. female smokers 11/36, *p* = 0.004); however, there was no difference in smoking pack years between sexes. Study participants had a variety of diagnoses including: chronic obstructive pulmonary disease (COPD) (*n* = 18), asthma (*n* = 25), interstitial lung disease (ILD) (*n* = 24), bronchiectasis (*n* = 5), pulmonary hypertension (*n* = 2), cardiac disease (*n* = 2), post-COVID-19 dyspnea (*n* = 1), and pneumothorax (*n* = 1). There was no difference in the proportion of male and female participants within the most frequent diagnosis groups (COPD, asthma, and ILD).

**TABLE 1:** Participant demographic, anthropomorphic, pulmonary function, dyspnea and symptom scores.

Variable	Males	Females
**Demographics**		
Participants, n	42	36
Age, years	64.1 ± 17.1	60.4 ± 15.6
Height, cm	175.3 ± 8.5	161.3 ± 7.6^†^
Mass, kg	87.3 ± 18.9	73.3 ± 20.8*
BMI, kg/m^2^	28.3 ± 5.0	28.0 ± 7.0
Pack years	21.5 ± 22.7	19.9 ± 30.8
**Pulmonary function**		
FEV_1_, L	2.49 ± 0.86 (*n* = 41)	1.93 ± 0.61* (*n* = 35)
FEV_1_,% predicted	80 ± 25	82 ± 18
FVC, L	3.49 ± 1.10	2.55 ± 0.72^†^
FVC,% predicted	86 ± 22	87 ± 18
FEV_1_/FVC	0.72 ± 0.14	0.76 ± 0.11
TLC, L	5.96 ± 1.84 (*n* = 25)	4.16 ± 1.18^†^ (*n* = 17)
TLC,% predicted	83 ± 24	80 ± 20
D_*LCO*_, mL.min^1^.mmHg^1^	16.4 ± 6.0	14.3 ± 4.6
D_*LCO*_,% predicted	63 ± 21 (*n* = 29)	71 ± 18 (*n* = 18)
**Dyspnea and symptom scores**		
mMRC, 0–4 scale	1.2 ± 1.0	1.6 ± 1.0
BDI total, 0–12 score	8.7 ± 2.6	7.7 ± 2.4
BDI task	2.5 ± 1.1	1.9 ± 1.1*
BDI effort	2.9 ± 1.0	2.8 ± 1.0
BDI impairment	3.3 ± 0.9	3.0 ± 0.8
MDP breathing discomfort, 0–10	4.7 ± 2.5	6.3 ± 1.9*
MDP immediate perception domain, 0–10	3.8 ± 2.3	5.3 ± 2.0*
MDP emotional response domain, 0–10	2.6 ± 2.4	4.1 ± 2.2*
HADS total	8.7 ± 5.5	11.4 ± 7.3
HADS depression	3.8 ± 3.1	4.6 ± 3.7
HADS anxiety	4.9 ± 3.5	6.9 ± 4.3*

Presented values are mean ± standard deviation, unless otherwise specified.

*p < 0.05;

^†^ *p* < 0.001. BDI, baseline dyspnea index; BMI, body mass index; D_*LCO*_, diffusing capacity of the lungs for carbon monoxide; FEV_1_, forced expiratory volume in 1 s; FVC, forced vital capacity; HADS, Hospital Anxiety and Depression Scale; MDP, Multidimensional dyspnea profile; mMRC, modified Medical Research Council dyspnea scale; TLC, total lung capacity.

Pulmonary function data are presented in Table 1. Males had greater absolute values for forced expiratory volume in 1 s (FEV_1_), forced vital capacity (FVC), and total lung capacity (TLC). However, when expressed as percentages of predicted values (29–31), males and females were similarly matched in terms of relative impairments in pulmonary function.

### 3.2 Dyspnea intensity, quality, and affective response

**FIGURE 1:**
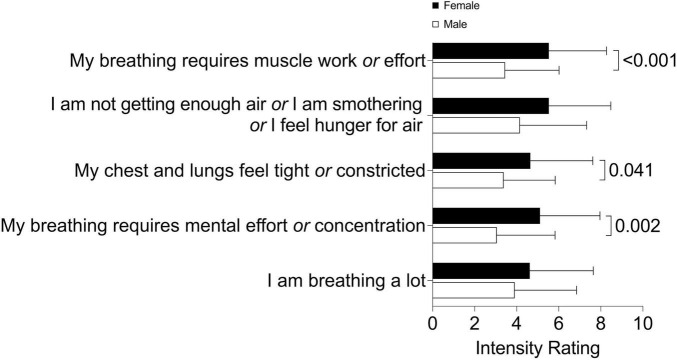
Multidimensional dyspnea profile (MDP) sensory dimension intensity ratings in males and females.

**FIGURE 2:**
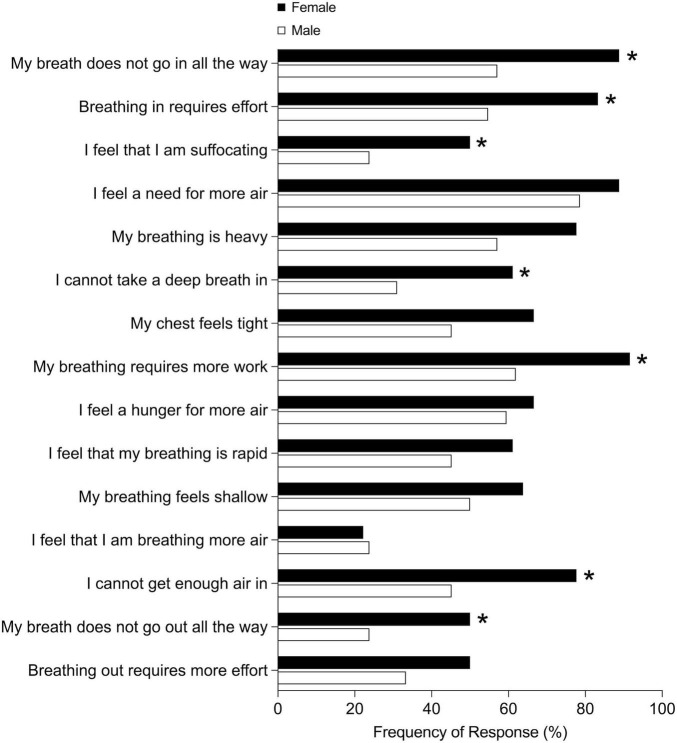
Selection of dyspnea quality descriptors in males and females. *p < 0.05.

**FIGURE 3:**
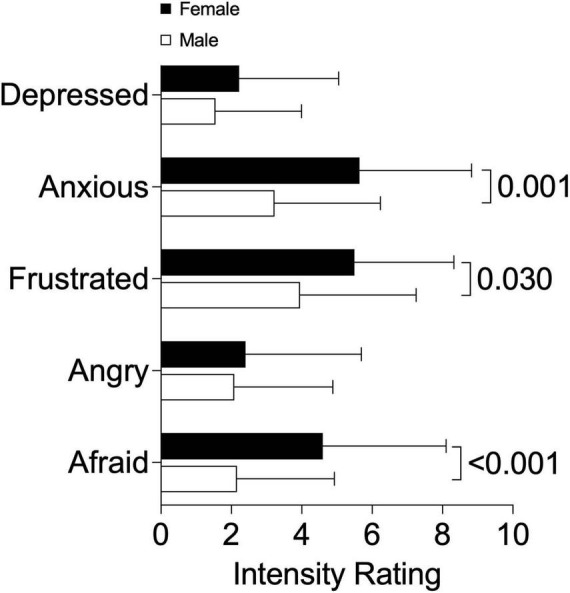
Multidimensional dyspnea profile emotional response intensity ratings in males and females.

Dyspnea intensity and symptom impact, assessed using the mMRC and BDI total scores, were similar between males and females (Table 1). However, females reported greater breathing discomfort and intensity of breathing sensations in the MDP immediate perception domain compared to males (Table 1). Females also rated higher intensity of muscle work or effort, chest tightness, and mental effort or concentration according to the MDP (Figure 1). Females had a greater selection frequency of dyspnea descriptors related to breathing work and effort, inspiratory difficulty, expiratory difficulty, and suffocating from the list of 15 dyspnea descriptors (14) (Figure 2). The affective response to dyspnea, reflected in the MDP emotional response domain, was also greater in females (Table 1), with significantly higher reports of anxiety, frustration, and fear related to dyspnea (Figure 3).

Sex, age, body mass index, and smoking status were significantly associated with breathing discomfort assessed using the MDP (F(5,70) = 3.90, *p* = 0.004). However, the proportion of variance explained by the model was relatively small (*R*^2^ = 0.22 and adjusted *R*^2^ = 0.16). Sex and HADS total score were the only significant independent variables in the regression model (Table 2).

**TABLE 2:** Multiple linear regression for breathing discomfort assessed using the MDP.

		95% CI for *B*					
Breathing discomfort	*B*	LL	UL	SE *B*	β	*R* ^2^	Δ*R*^2^
Model						0.22	0.16
Constant	3.95	0.63	7.28	1.67			
Sex	−1.51	−2.57	−0.44	0.54	−0.32		
Age	−0.01	−0.04	0.03	0.02	−0.07		
BMI	0.06	−0.02	0.14	0.04	0.15		
Smoking status	0.31	−0.26	0.89	0.29	0.13		
HADS total	0.09	0.01	0.18	0.04	0.24		

B, unstandardized regression coefficient; β, standardized coefficient; CI, confidence interval; LL, lower limit; MDP, Multidimensional Dyspnea Profile; *R*^2^, coefficient of determination; Δ*R*^2^, adjusted R^2^; SE *B*, standard error of the coefficient; UL, upper limit.

### 3.3 Anxiety and depression

Females had higher HADS total and anxiety subscale scores compared to males (Table 1). The proportion of female participants with a HADS anxiety score equal to or greater than 8 was not significantly different compared to males (males 12/42 vs. females 17/36, *p* = 0.105). There was no significant difference in HADS depression subscale scores between males and females. There was no difference in the proportion of male and female participants with HADS depression subscale scores in the lower versus higher likelihood groups (threshold ≥ 8 to define higher likelihood group).

Completion of the VAS rating of comfort discussing feelings of anxiety with a physician was challenging for many participants to perform on Zoom. A subset (*n* = 30) of participants with HADS depression and anxiety scores representative of the entire sample were able to provide a VAS scale rating. Both males and females felt comfortable discussing anxiety (males 0.93 ± 0.15 vs. females 0.93 ± 0.08, *p* = 0.958).

### 3.4 Relationships between dyspnea and anxiety

Exploratory analysis assessed correlations between dimensions and domains of the MDP and HADS anxiety subscale scores. In male participants, higher HADS anxiety subscale score was significantly correlated with higher MDP immediate perception domain (*r* = 0.519, *p* < 0.001), emotional response domain (*r* = 0.652, *p* < 0.001), and breathing discomfort (*r* = 0.524, *p* < 0.001). However, in female participants, higher HADS anxiety subscale score was only correlated with the emotional response domain (*r* = 0.432, *p* = 0.009), and not with the immediate perception domain (*r* = 0.257, *p* = 0.131) nor breathing discomfort (*r* = 0.058, *p* = 0.736).

**FIGURE 4:**
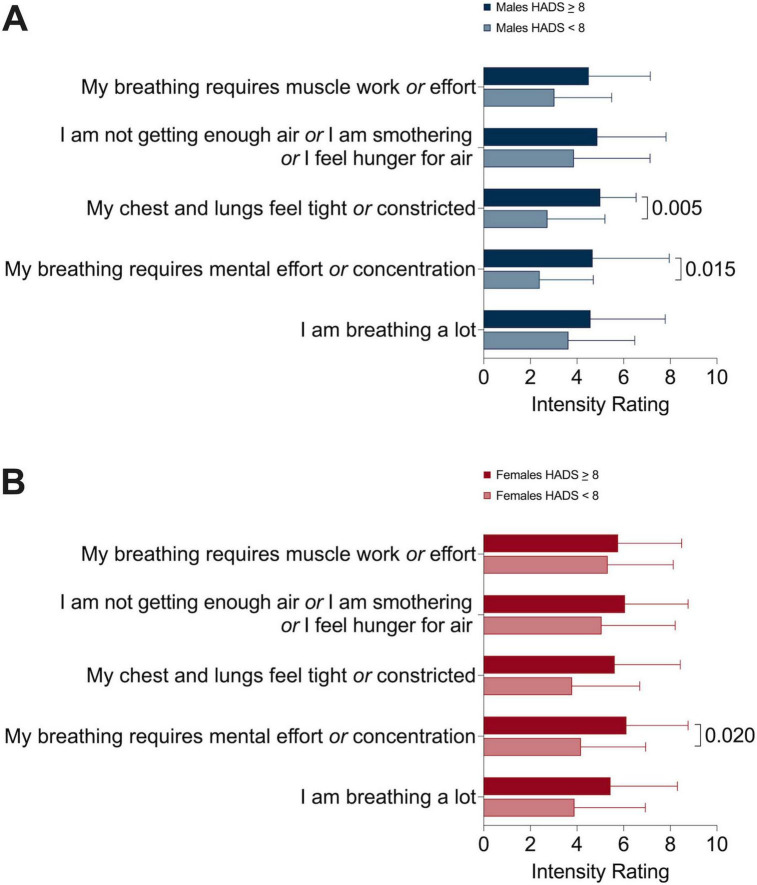
Multidimensional dyspnea profile sensory dimension intensity ratings in **(A)** males and **(B)** females comparing higher versus lower likelihood of anxiety sub-groups.

**FIGURE 5:**
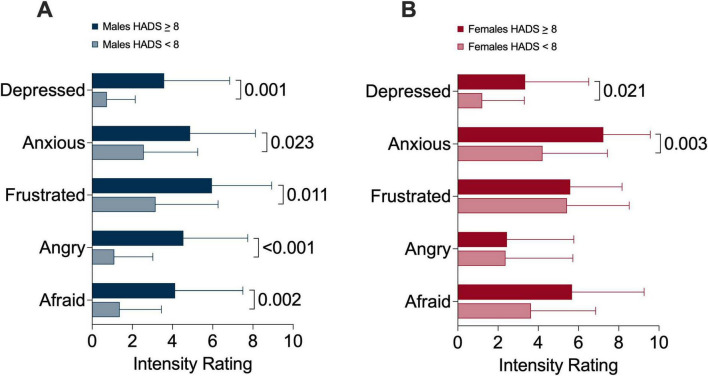
Multidimensional dyspnea profile emotional response intensity ratings in **(A)** males and **(B)** females comparing higher versus lower likelihood of anxiety sub-groups.

Male and female participants were stratified based on a higher versus lower likelihood of anxiety and intensity of sensory qualities of dyspnea (Figure 4) and emotions related to dyspnea (Figure 5) and were compared within sexes. In both sexes, participants with higher anxiety scores experienced greater mental breathing effort compared to their counterparts with lower anxiety scores (Figures 4A, B). Males with higher anxiety scores experienced greater intensity of frustration, anger, and fear related to dyspnea (Figure 5A); however, this was not the case for females (Figure 5B).

## 4 Discussion

We examined sex-differences in dyspnea intensity, sensory perception, and emotional impact in respiratory outpatients. Our study population was reflective of a real-world outpatient respiratory medicine setting and included participants with a variety of respiratory diagnoses including COPD, asthma, and ILD. In this group of males and females with similar relative lung function impairment and mMRC dyspnea scores, females experienced greater physical and mental breathing effort as well as greater chest tightness compared to males. In a regression model, sex and HADS total score were significant independent variables. However, the proportion of variation in breathing discomfort explained by the model was low, highlighting that determinants of dyspnea are complex. Increased work and effort, inspiratory and expiratory difficulty, and suffocating sensations were more frequently selected by females. Our results are novel in demonstrating that despite similar lung function impairment and dyspnea intensity based on the commonly used mMRC questionnaire, females experience significantly greater anxiety, frustration, and fear related to dyspnea. These results highlight that dyspnea quality and emotional responses are unique in females and underscores the importance of multidimensional dyspnea assessment.

Greater dyspnea intensity in females has been examined in several studies and is proposed to be related, at least in part, to lower absolute lung volumes in females compared to males (2, 3). During exertion, this difference in absolute lung volume results in females operating at a higher proportion of ventilatory capacity for any given absolute workload (7, 10, 11, 32). Consequently, females experience greater dyspnea intensity at matched absolute ventilation or work rate compared to males (7, 10, 11, 32). Distinct sex-differences in dyspnea quality have been investigated in healthy and clinical populations. Physically active young females select qualitative descriptors of dyspnea related to unsatisfied inspiration and shallow breathing more often than their male counterparts at peak exercise (7). Previous studies have identified that females frequently select qualitative descriptors of breathing work and effort, inspiratory difficulty, expiratory difficulty, and suffocating, similar to the results of this study (7, 10, 14). This suggests systematic underpinnings to observed sex-differences in dyspnea quality across health and disease.

The most well-understood sex-difference in exertional dyspnea quality is the sensation of unsatisfied inspiration. This sensation is thought to be related to females encroaching on critical inspiratory mechanical constraints relatively earlier during exercise compared to males (10, 33). Importantly, operating lung volumes during exercise are similar between males and females when expressed relative to ventilation as a fraction of maximum ventilatory capacity (7, 10). This again underscores the importance of sex-differences in absolute lung volumes, which underpins not only differences in dyspnea intensity but also distinct aspects of dyspnea quality. Our study extends these observations to a clinical outpatient respirology population, demonstrating that females experience heightened sensations of breathing work and effort, as well as greater inspiratory difficulty.

Structured assessment of the affective response to dyspnea in individuals with chronic respiratory disease is under reported in the literature. We observed that females experience significantly more anxiety, frustration, and fear related to dyspnea compared to males. Anxiety, depression, and breathlessness frequently co-exist in the general population and are associated with reduced functional status (25). Females in our study had higher HADS total and anxiety subscale scores, although the proportion of participants with higher likelihood of anxiety was not different between groups. We found that higher HADS anxiety subscale scores were correlated with MDP dyspnea scores of breathing discomfort, immediate perception domain, and emotional response domain in males. However, in females, higher HADS anxiety scores were correlated with the emotional response domain only. This is similar to results of a study performed in healthy individuals where anxiety scores were correlated with work and effort ratings of dyspnea in males but not in females (34). These findings suggest that the inter-relationships between dyspnea, the affective response to dyspnea, and anxiety may be different between sexes.

We further explored differences between higher versus lower likelihood of anxiety within male and female groups. Interestingly, we demonstrated that frustration, anger, and fear related to dyspnea was not different based on the likelihood of anxiety in females; however, this was the case for male participants. Taken together, although females as a group had higher mean HADS anxiety subscale scores, anxiety scores were correlated with multiple aspects of dyspnea in males but not females. Elucidating the reasons for this observation extends beyond the scope of our study; however, our findings suggest that anxiety may not interact with different dimensions of dyspnea in a uniform way in males and females, or that additional variables could underpin or modify the higher anxiety, frustration, and fear related to dyspnea that females experience.

There are several limitations to our study that must be acknowledged. Participants rated multidimensional aspects of dyspnea based on the worst episode of dyspnea they had experienced in the preceding 6 weeks and it is possible that recall bias impacted our results. Although the MDP has been used in previous studies for periods up to 6 weeks (35), reliability of recall diminishes over time, which may have negatively impacted the reliability of our results. Future studies will be needed to prospectively assess sex-differences in dyspnea intensity and quality over time. In addition, our study presents results from respiratory outpatients with a variety of diagnoses, and as such, we were not sufficiently powered to determine if our observations of the whole group were true within disease subgroups. This limits our ability to draw conclusions about sex-differences between diseases, but increases the generalizability of our results to a real world, mixed diagnosis population encountered in outpatient respirology practice. Future work involving a larger study population will be needed to examine potential variations in sex-differences in dyspnea between disease groups.

Previous studies have predominantly focused on sex-differences in dyspnea intensity and the relationship to lung function and exercise physiology responses. This has been crucial in informing an understanding of sex-differences in exertional dyspnea intensity. This study builds upon previous observed sex-differences in dyspnea quality and describes greater anxiety, frustration, and fear related to dyspnea in females. Although higher anxiety scores were moderately correlated with multiple dimensions of dyspnea in males, this was not the case in females. Likelihood of anxiety also did not differentiate between emotional responses of frustration, anger, and fear in females, although this was the case for males. Understanding sex-differences in affective responses to dyspnea requires further investigation. The sex-differences in dyspnea quality and affective response among respiratory outpatients with similar mMRC dyspnea scores, highlights the importance of multidimensional dyspnea assessment in clinical settings. The lived patient experience of diverse dyspnea qualities and negative emotional responses would be easily underappreciated when relying on a unidimensional assessment of dyspnea alone.

## Data Availability

The raw data supporting the conclusions of this article will be made available by the authors, without undue reservation.
